# Whole-Genome Sequencing Demonstrates That Fidaxomicin Is Superior to Vancomycin for Preventing Reinfection and Relapse of Infection With *Clostridium difficile*

**DOI:** 10.1093/infdis/jit598

**Published:** 2013-11-11

**Authors:** David W. Eyre, Farah Babakhani, David Griffiths, Jaime Seddon, Carlos Del Ojo Elias, Sherwood L. Gorbach, Tim E. A. Peto, Derrick W. Crook, A. Sarah Walker

**Affiliations:** 1 NIHR Oxford Biomedical Research Centre, University of Oxford, United Kingdom; 2 Optimer Pharmaceuticals, San Diego, California

**Keywords:** *Clostridium difficile*, recurrence, fidaxomicin, whole genome sequencing

## Abstract

Whole-genome sequencing was used to determine whether the reductions in recurrence of *Clostridium difficile* infection observed with fidaxomicin in pivotal phase 3 trials occurred by preventing relapse of the same infection, by preventing reinfection with a new strain, or by preventing both outcomes. Paired isolates of *C. difficile* were available from 93 of 199 participants with recurrences (28 were treated with fidaxomicin, and 65 were treated with vancomycin). Given *C. difficile* evolutionary rates, paired samples ≤2 single-nucleotide variants (SNVs) apart were considered relapses, paired samples >10 SNVs apart were considered reinfection, and those 3–10 SNVs apart (or without whole-genome sequences) were considered indeterminate in a competing risks survival analysis. Fidaxomicin reduced the risk of both relapse (competing risks hazard ratio [HR], 0.40 [95% confidence interval {CI}, .25–.66]; *P* = .0003) and reinfection (competing risks HR, 0.33 [95% CI, 0.11–1.01]; *P* = .05).

Fidaxomicin is a promising new agent for the treatment of healthcare-associated infection with *Clostridium difficile*. Compared with the previous standard-of-care, vancomycin, fidaxomicin is associated with a lower incidence of *C. difficile* infection (CDI) recurrence, as demonstrated in 2 phase 3 trials [1, 2], and with a decreased risk of the composite end point of persistent diarrhea, recurrence, or death, according to a meta-analysis [3]. However, 16%–50% of CDI recurrences are actually reinfections with a different strain [4], and 83% of recurrences in the phase 3 trials involved *C. difficile* strains with the same restriction endonuclease analysis type [5]. Whether benefits from fidaxomicin derive from preventing relapse of the same infection or preventing reinfection shortly after clearance of the initial infection has not been previously investigated.

Several methods can be used to distinguish same-strain relapse from new-strain reinfection. Traditional genotyping methods such as ribotyping or multilocus sequencing typing may not detect diversity present at a whole-genome level [6] and may therefore underestimate reinfection rates, particularly where strains are common (eg, NAP1/ribotype-027/ST1) or diverse (eg, ribotype-015).

Our objective was therefore to use data from the original phase 3 trials and whole-genome sequencing to estimate the impact of fidaxomicin versus vancomycin on *C. difficile* same-strain relapse versus new-strain reinfection.

## METHODS

Both multicenter, double-blind trials followed the same protocol and randomly assigned adults with CDI to received fidaxomicin 200 mg twice daily or vancomycin 125 mg 4 times daily for 10 days [1, 2]. Participants were ≥18 years old; had >3 unformed bowel movements in the 24 hours before randomization; had *C. difficile* toxin A, toxin B, or both detected in stool; had received ≤4 doses of vancomycin or metronidazole for a total period of ≤24 hours; and had ≤1 CDI episode in the previous 3 months. *C. difficile* toxin testing was performed locally (mostly by enzyme immunoassays). Both trials received ethics approval at all centers, and all participants provided written informed consent.

For both trials, the primary end point was clinical cure defined as resolution of diarrhea (determined by ≤3 unformed stools for 2 consecutive days) maintained for the subsequent duration of therapy with no further CDI therapy required before the end-of-therapy assessment, performed 2 days after the end of the 10-day treatment course. Participants with clinical cure were followed for a further 28 days to ascertain CDI recurrence. Here, we consider the end point of CDI recurrence in an intention-to-treat (ITT) analysis that included all randomized patients with clinical cure, counting time from the end of the treatment course.

Isolates were sequenced using Illumina HiSeq 2000 (Illumina, San Diego, CA), generating 100 base-pair reads. Properly paired reads were mapped using Stampy, version 1.0.17 (without Burrows-Wheeler Aligner premapping, using an expected substitution rate of 0.01), to the *C. difficile* 630 reference genome, CD630 (GenBank accession AM180355.1). Single-nucleotide variants (SNVs) were identified across all mapped nonrepetitive sites, using mpileup (options -E -M0 –Q25 -q30 -m2 -D –S) in SAMtools, version 0.1.18. Repetitive regions were identified by performing Basic Local Alignment Search Tool searches of the reference genome, using fragments of the same genome. We only used SNVs supported by ≥5 reads, including 1 in each direction, and a consensus of ≥90% high-quality bases (Phred quality score, ≥25). Calls required ≥35% of bases in reads spanning a SNV to have a Phred quality score of ≥25. A median of 84% (interquartile range, 83.8%–84.8%) of the CD630 reference genome was called across all isolates. Sequencing analysis was conducted blinded to treatment regimen.

Recurrent CDI could involve (1) the same strain as that present at randomization, ie a relapse of the original infection (2) a different strain from that present at randomization, ie a reinfection or (3) an unknown strain, for example where an isolate was missing or culture negative, making it impossible to distinguish between (1) and (2). Recurrent infections involving an isolate with ≤2 SNVs from the initial isolate were defined as relapses, those involving an isolate with >10 SNVs from the initial isolate were defined as reinfections, and those involving an isolate with 3–10 SNVs from the initial isolate or for which both paired isolates were not available (including infections for which no isolate could be cultured from the original or follow-up sample) were defined as indeterminate [7]. Within the study, observing a “relapse” means that a “reinfection” can no longer be observed (and vice versa); we therefore used competing risks survival analysis [8] to compare the impact of fidaxomicin versus vancomycin on the cumulative incidence of the different types of recurrence. Results obtained by cause-specific hazard regression were very similar to those obtained by completing risks survival analysis (data not shown).

## RESULTS

A total of 1164 participants were enrolled in the 2 trials [1, 2], of whom 990 experienced clinical cure and were assessed for recurrence. Overall, in the ITT analysis, 68 of 494 (14%) randomly assigned to receive fidaxomicin, compared with 131 of 496 (26%) randomly assigned to receive vancomycin, experienced CDI recurrence (hazard ratio [HR], 0.46 [95% confidence interval {CI}, .35–.62]; *P* < .001]). Isolates were obtained at the times of randomization and recurrence from 93 of 199 participants (47%) and stored; these isolates were from 28 of 68 fidaxomicin recipients (41%) with CDI recurrence compared with 65 of 131 vancomycin recipients (50%) with CDI recurrence (*P* = .29). Participants with and those without stored isolates were similar in terms of strain, creatinine level, albumin level, and white blood cell count but were more likely to be younger, to be from Canada [5], to be outpatients, to have mild or severe disease, to have a higher hematocrit, and to have more unformed stools (Supplementary Table 1). Missing isolates were never stored (for 91 patients [46%]) or specimens were culture negative (for 15 [8%]) at baseline only (9 and 5 isolates, respectively), at recurrence only (60 and 7, respectively), or at both time points (22 and 3, respectively). Whole-genome sequences were obtained from all 93 recurrences with stored paired isolates. A total of 43 baseline strains (46%) were from clade 2 (36 ribotype-027/ST1), 45 (48%) were from the large heterogeneous clade 1 (16 different STs), 2 were from clade 4 (ribotype-017/ST37), 2 were from clade 5 (ribotype-078/ST11), and 1 was from clade 3 (ribotype-023/ST5).

**Figure 1.**
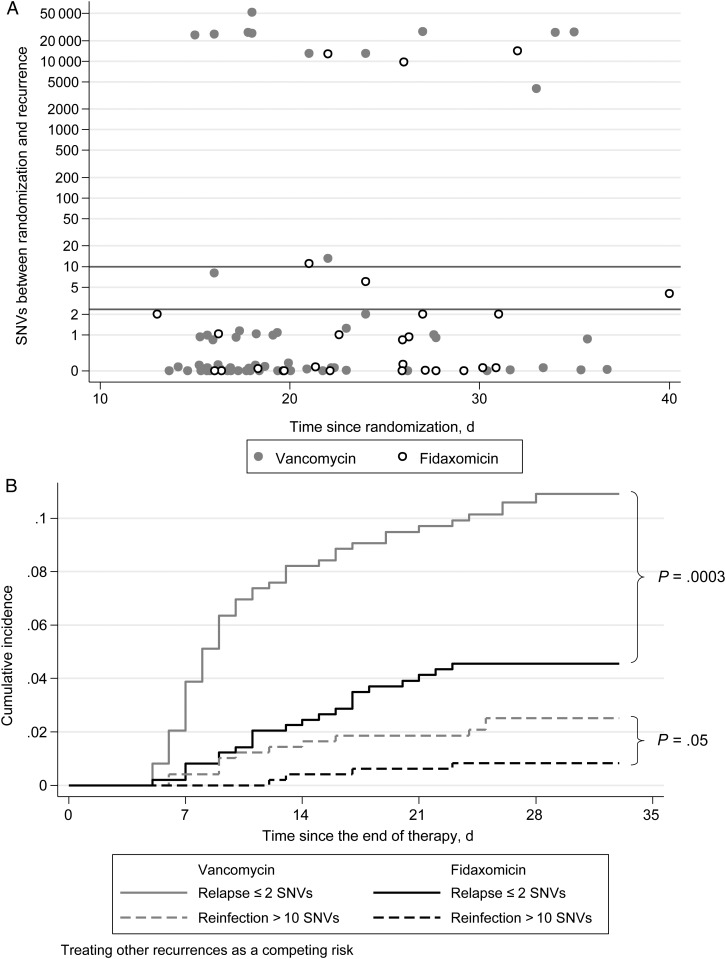
Single-nucleotide variants (SNVs) between randomization and recurrence (*A*) and cumulative incidence of relapse and reinfection (*B*). In panel *A*, a small amount of jitter has been added to points with 0 and 1 SNVs to enable visualization. In panel *B*, *P* values are for competing risks hazard ratios.

For 54 of 93 participants (58%), there were 0 SNVs between the isolate recovered at randomization and the isolate recovered during recurrence. Paired isolates from 16 participants (17%) had 1 SNV; those from 4 (4%) had 2 SNVs; those from 1 each had 4, 6, 8, 11, and 13 SNVs; and those from 14 (15%) had ≥3970 SNVs (Figure 1*A*). Of the recurrent isolates with ≥3970 SNVs from the baseline isolates, 9 were clade 1 strains, and 5 were clade 2 strains. Based on estimated evolutionary rates of 0.74 SNVs/called-genome/year (95% CI, .22–1.40) and mean within-host diversity of 0.30 SNVs (95% CI, 0.13–0.42) [7], between 0 and 2 SNVs would be expected between relapse isolates recovered up to 40 days apart (95% prediction interval). Interestingly, the participants with 4, 8, 11, and 13 SNVs between isolates were all from the eastern United States or Canada, were infected with ribotype-027/ST1 at both randomization and recurrence, and had SNVs between the paired isolates distributed throughout the genome. Three participants (those with 8, 11, and 13 SNVs between isolates) had no prior CDI within the 3 months before study enrollment. The highly prevalent ribotype-027/ST1 strain has less within-genotype diversity, possibly because of its recent emergence [6], suggesting that these SNVs are more likely to have arisen via reinfection rather than from within-host microevolution, which does not differ significantly between ribotype-027 and other lineages [6]. The participant with 6 SNVs between isolates was infected with ribotype-001/072/ST3 at both time points.

Classifying same-strain relapse as a follow-up isolate with ≤2 SNVs from the baseline isolate and reinfection as a follow-up isolate with >10 SNVs from the baseline isolate, 22 of 494 fidaxomicin recipients (4%) and 52 of 496 vancomycin recipients (10%) experienced relapse (competing risks HR, 0.40 [95% CI, .25–.66]; *P* = .0003), and 4 (0.8%) and 12 (2%), respectively, had reinfection (competing risks HR, 0.33 [95% CI, 0.11–1.01]; *P* = .05; Figure 1*B*). In 42 fidaxomicin recipients (9%) and 67 vancomycin recipients (14%), recurrence could not be classified because of a missing isolate or indeterminate SNVs (competing risks HR, 0.59 [95% CI, 0.40–0.87]; *P* = .007). Similar results were obtained using a >2 SNV threshold for reinfection and in the modified ITT population [1, 2] (data not shown). The effect of fidaxomicin compared with vancomycin remained similar after adjustment for important baseline cofactors, including age, past CDI, number of unformed bowel movements, albumin level, white blood cell count, and creatinine level (relapse *P* < .001; reinfection *P* < .07).

Each of the 20 patients with 1–2 SNVs between baseline and follow-up isolates developed different SNVs (Table 1). Across all 20 patients, 24 SNVs were detected, 19 in coding sequences, resulting in 4 synonymous changes (ie no amino acid changes) and 15 nonsynonymous changes (ie amino acid changes) including a single truncation (dN/dS ratio, 1.3). *RpoB* was the only gene affected by >1 mutation; adjacent SNVs within the same codon occurred in 2 patients receiving fidaxomicin. *rpoB* codes for the β subunit of a DNA-directed RNA polymerase, which contains a fidaxomicin binding site. One mutation, previously reported and detected in this isolate by capillary sequencing, resulted in a Val1143Gly substitution. The initial patient isolate had a fidaxomicin minimum inhibitory concentration (MIC) of 0.06 µg/mL, and the subsequent isolate with the Val1143Gly substitution had a fidaxomicin MIC of 16 µg/mL [9]. The second SNV caused a Val1143Leu substitution and an increase in MIC from 0.015 µg/mL to 0.125 µg/mL at follow-up. Other non-synonymous mutations included a SNV in *spo0A*, a stage 0 sporulation protein.

**Table 1.** List of Single Nucleotide Variants (SNVs) and Affected Genes in 20 Patients With *Clostridium difficile* Infection (CDI) Relapse and 1–2 SNVs

Baseline Sample	Recurrence Sample	SNV Site	Baseline	Recurrence	Locus Tag	Effect	Gene	Function	Treatment Arm
C00011058	C00011057	1 529 772	C	T	CD1319	Synonymous	*…*	Putative polysaccharide deacetylase	Fidaxomicin
C00011030	C00011031	324 403	A	G	CD0263	Synonymous	*flhA*	Flagellar biosynthesis protein A	Vancomycin
C00010896	C00010897	3 480 557	G	T	CD2996	Nonsynonymous	*…*	Hypothetical protein	Vancomycin
C00010975	C00010976	2 936 847	G	A	CD2540	Nonsynonymous	*nox*	Coenzyme A disulfide reductase	Vancomycin
**C00010958**	**C00010957**	**93 587**	**T**	**G**	**CD0066**	**Nonsynonymous**	***rpoB***	**DNA-directed RNA polymerase subunit beta**	**Fidaxomicin**
C00010993	C00010991	1 853 475	G	T	CD1597	Nonsynonymous	*…*	Hypothetical protein	Vancomycin
C00010949	C00010948	2 747 475	C	A	CD2381	Nonsynonymous	*iorA*	Indolepyruvate oxidoreductase subunit	Vancomycin
C00011003	C00011002	2 618 834	The site 2618834 is not in a coding region						Vancomycin
C00010995	C00010994	1 858 469	C	A	CD1604	Synonymous	…	ABC transporter, ATP-binding protein	Vancomycin
C00010894	C00010893	4 202 583	The site 4202583 is not in a coding region						Fidaxomicin
C00011014	C00011015	3 786 856	A	G	CD3234	Nonsynonymous	…	Hypothetical protein	Vancomycin
C00010915	C00010916	1 203 029	G	T	CD1030	Nonsynonymous	*…*	Putative glycosyl transferase	Vancomycin
C00011017	C00011016	1 318 964	G	A	CD1121	Truncation	*dhaB2*	Glycerol dehydratase activator	Vancomycin
C00010926	C00010927	2 460 963	A	G	CD2125	Synonymous	*…*	Putative acetyltransferase	Vancomycin
C00011075	C00011074	862 374	A	G	CD0707	Nonsynonymous	*…*	Putative signaling protein	Vancomycin
C00011037	C00011036	1 412 733	C	T	CD1214	Nonsynonymous	*spo0A*	Stage 0 sporulation protein A	Fidaxomicin
		2 676 088	G	A	CD2314	Nonsynonymous	…	Hypothetical protein	
C00010910	C00010909	716 438	The site 716438 is not in a coding region						Fidaxomicin
		2 268 602	The site 2268602 is not in a coding region						
C00011033	C00011032	1 079 113	G	A	CD0896	Nonsynonymous	…	Hypothetical protein	Fidaxomicin
		2 615 540	A	T	CD2256	Nonsynonymous	…	Putative PTS system, EIIc component	
C00011006	C00011007	2 079 466	The site 2079466 is not in a coding region						Vancomycin
		4 167 337	G	A	CD3566	Nonsynonymous	*ipk*	4-diphosphocytidyl-2-C-methyl-D-erythritol kinase	
**C00010895**	**C00016336**	**93 586**	**G**	**C**	**CD0066**	**Nonsynonymous**	***rpoB***	**DNA-directed RNA polymerase subunit beta**	**Fidaxomicin**

Two mutations affecting the *rpoB* gene are highlighted in bold. A total of 54 patients had a relapse of CDI with 0 SNVs between baseline and recurrence samples.

## DISCUSSION

This study demonstrates that fidaxomicin protects against CDI recurrence by preventing both relapse of the initial infection and reinfection with a new strain. Fidaxomicin was associated with a 2.5-fold lower cumulative risk of relapse and 3-fold lower cumulative risk of reinfection up to 28 days after therapy, compared with vancomycin.

The protective effect of fidaxomicin may be mediated by less disruption of the intestinal microbiome, compared with vancomycin [10]. Greater preservation of normal bowel flora may inhibit both relapse and new infections. In vitro CDI gut model studies also suggest that fidaxomicin becomes sequestered into biofilms and adheres to spores and therefore may also offer an advantage by remaining active in the gut for longer than vancomycin [11].

Whole-genome sequencing of isolates allowed precise identification of same strain relapses and reinfections, even among NAP1/BI/ribotype-027 strains. Although in the main analysis we conservatively defined differences of 3–10 SNVs between baseline and recurrence isolates as indeterminate, these cases are more likely to represent reinfection, given rates of *C. difficile* evolution: even with an evolution rate of 2 SNVs/called-genome/year and a mean of 0.3 SNVs from baseline diversity, 2 SNVs remains the upper limit of the 95% prediction interval over the timescale of this study. Supporting this, all but one of the likely reinfecting strains with 3–10 SNVs were from the highly prevalent NAP1/BI/ribotype-027 lineage, and the last was from a sequence type (ST3) that includes substantial diversity [6]. Classing all subsequent isolates with >2 SNVs from the baseline isolate as reinfection produced similar results to the main analysis, and our choice of the 2 and 10 SNV thresholds was made on the basis of previous work [7], rather than during this study. The number of reinfections highlights the contribution of ongoing exposure to *C. difficile* to disease recurrence and the potential for focused infection control efforts to prevent new transmissions to CDI cases, as well as onward transmission from CDI cases. As observed previously [4], the timing of relapses and reinfections overlap considerably (Figure 1*A*), requiring the use of genotyping or, preferably, highly discriminatory sequencing or multilocus variable number tandem repeat analysis to distinguish these 2 scenarios.

The *rpoB* gene mutations arising de novo in 2 patients receiving fidaxomicin conferred reduced susceptibility to fidaxomicin, although susceptibility reductions were modest in 1 patient. The Val1143Gly mutation has been previously described in spontaneous single-step mutagenesis studies [12]. The extent to which these mutations contributed to relapse in these patients is unclear, as the MICs remained well within the range thought to be effective (typical fidaxomicin fecal concentrations are approximately 1000 µg/g) [12]. Neither mutation was observed in either baseline or recurrence isolates in any other patient, suggesting they are uncommon.

The main study limitation is that paired isolates were only stored for 93 of 199 recurrences; as previously reported, stored samples were more likely to be from younger patients and from Canadian sites [5], as well as from outpatients and participants with more unformed bowel movements and higher hematocrits. However, because storage was done when participants and healthcare workers were blinded to randomized allocation, because there was no other systematic bias in which samples were stored and sequenced, and because patients were randomly assigned to receive fidaxomicin or vancomycin (so effect estimates cannot be confounded), the benefit from fidaxomicin should be robust. Another limitation is generalizability outside of the trial population studied; although the entry criteria for both trials were relatively broad, participants were, for example, younger than typical individuals with CDI. However, benefits from fidaxomicin versus vancomycin on recurrence overall were similar in multiple subgroups [3], suggesting our findings should be generalizable. Only single colony picks were stored and sequenced, so it is possible that variants identified in relapses could have been present previously but not sampled [13]. Perhaps more importantly, apparent reinfections could theoretically be the result of sampling an initially mixed infection. However, such a bias in identifying relapse versus reinfection should apply equally to both randomized groups and should not therefore impact the estimated benefits of fidaxomicin compared with vancomycin. Storing stool specimens and sequencing mixed growth from primary or subculture plates would enable specific bioinformatic tools to be used to detect this in future studies [14].

In summary, we have shown that fidaxomicin prevents relapse and short-term reinfection. It is therefore likely to benefit patients at high risk of recurrence [15] and also those at high risk of reexposure, such as individuals in an outbreak setting. Whole-genome sequencing was able to detect mutations conferring reduced susceptibility to fidaxomicin and may therefore be a valuable tool in *C. difficile* antimicrobial resistance surveillance.

## Supplementary Data

Supplementary materials are available at *The Journal of Infectious Diseases* online (http://jid.oxfordjournals.org/). Supplementary materials consist of data provided by the author that are published to benefit the reader. The posted materials are not copyedited. The contents of all supplementary data are the sole responsibility of the authors. Questions or messages regarding errors should be addressed to the author.

Supplementary Data
