# Supplementary material for: Whole-Genome Sequencing Demonstrates That Fidaxomicin Is Superior to Vancomycin for Preventing Reinfection and Relapse of Infection With Clostridium difficile
Source: J Infect Dis. 2013 Nov 11;209(9):1446–51. doi: 10.1093/infdis/jit598 (PMC3982846; doi:10.1093/infdis/jit598)
Supplement: Supplementary Data [file supp_209_9_1446__index.html]

Whole-Genome Sequencing Demonstrates That Fidaxomicin Is Superior to Vancomycin for Preventing Reinfection and Relapse of Infection With Clostridium difficile — Supplementary Data 

# Whole-Genome Sequencing Demonstrates That Fidaxomicin Is Superior to Vancomycin for Preventing Reinfection and Relapse of Infection With *Clostridium difficile*

## Supplementary Data

Supplementary Data

**Files in this Data Supplement:**

- Supplementary Data - Pdf file
