# Supplementary material for: Whole-Genome Sequencing Demonstrates That Fidaxomicin Is Superior to Vancomycin for Preventing Reinfection and Relapse of Infection With Clostridium difficile
Source: J Infect Dis. 2013 Nov 11;209(9):1446–51. doi: 10.1093/infdis/jit598 (PMC3982846; doi:10.1093/infdis/jit598)
Supplement: Supplementary Data [file supp_jit598_jit598supp.pdf]

Factor		No WGS data available*		WGS data available		Exact p / rank-sum p
Treatment Arm	Vancomycin	66	50%	65	50%	0.29
	Fidaxomicin	40	59%	28	41%	
**Country / Region**	**USA**	**57**	**63%**	**33**	**37%**	**<0.001**
	**Canada**	**29**	**36%**	**52**	**64%**	
	**Europe**	**20**	**71%**	**8**	**29%**	
Strain**	BI	27	43%	36	57%	0.40
	non-BI	31	36%	56	64%	
Prior CDI	No prior CDI	85	54%	72	46%	0.73
	Single prior CDI	21	50%	21	50%	
Gender	Female	56	50%	57	50%	0.25
	Male	50	58%	36	42%	
**Patient Status**	**Inpatient**	**78**	**63%**	**45**	**37%**	**<0.001**
	**Outpatient**	**28**	**37%**	**48**	**63%**	
**Severity**	**Mild**	**34**	**58%**	**25**	**42%**	**0.02**
	**Moderate**	**42**	**64%**	**24**	**36%**	
	**Severe**	**30**	**41%**	**44**	**59%**	
**Age**	**Median**	**70**		**63**		**0.01**
	**IQR**	**59 - 80**		**50 - 76**		
Albumin (g/L)	Median	29		30		0.11
	IQR	25 - 33		25 -37		
BUN (mmol/L)	Median	5.4		5		0.67
	IQR	3.9 - 7.9		3.9 - 8.6		
Creatinine (µmol/L)	Median	69		69		0.40
	IQR	53 - 99		53 - 99		
**Hematocrit (%)**	**Median**	**35**		**37**		**0.01**
	**IQR**	**32 - 38**		**33 - 41**		
WBC (10^9/L)	Median	9.1		8.7		0.96
	IQR	(7.4 - 12.3)		(6.2 - 13.4)		
Neutrophils (10^9/L)	Median	7		6.4		0.67
	IQR	5.2 - 9.5		4.2 10.3		
**Number of unformed bowel motions in 24 hours**	**Median**	**6**		**8**		**0.03**
	IQR	5-9		5 - 10		

Online Table 1. Characteristics of participants with and without whole genome sequence data. IQR, interquartile range. Factors with a significant association are shown in bold.

* one or both of baseline and subsequent recurrence isolate not stored (n=91) or culture-negative or culture-negative (n=15)

** strain by REA typing was missing for 50 recurrences without and 1 recurrence with WGS.
